# Phenotypical evaluation of lymphocytes and monocytes in patients with type 2 diabetes mellitus in Saudi Arabia

**DOI:** 10.15537/smj.2023.44.3.20220873

**Published:** 2023-03

**Authors:** Mamdouh Allahyani, Amani M. Alshalawi, Maram R. Alshalawii, Shahad A. Alqorashi, Abdulelah Aljuaid, Mazen M. Almehmadi, Mohammed A. Bokhary, Alhanouf S. Albrgey, Ahmad A. Alghamdi, Abdullah F. Aldairi, Ayman S. Alhazmi

**Affiliations:** *From the Department of Clinical Laboratory Sciences (Allahyani, Alshalawi, Alshalawii, Alqorashi, Aljuaid, Almehmadi, Alghamdi, Alhazmi), College of Applied Medical Sciences, Taif University, from the Department of Clinical Chemistry (Bokhar); from the Department of Endocrinology and Diabetic Centre (Albrgey), King Abdulaziz specialist hospital, Ministry of Health, Taif, and from the Department of Laboratory Medicine (Aldairi), Faculty of Applied Medical Sciences, Umm Al-Qura University, Makkah, Kingdom of Saudi Arabia.*

**Keywords:** lymphocytes, monocytes, T2DM

## Abstract

**Objectives:**

To evaluate the levels of total lymphocytes, B-lymphocytes (CD19+), T-lymphocytes (CD3+), natural killer (NK) cells (CD3-/CD56+), and monocyte subsets in type 2 diabetes mellitus (T2DM) patients in Saudi Arabia. In addition, this study aimed to evaluate whether B- and T-lymphocyte subsets are frequently altered in patients with T2DM.

**Methods:**

A case-control study included 95 participants recruited in the study: 62 patients with T2DM and 33 healthy individuals. All the patients were admitted to the Diabetic Centre in Taif, Saudi Arabia. Blood samples were collected between April and August 2022. The hemoglobin A1c (HbA1c) level was evaluated in all patients. Flow cytometry was used to measure the expression of B-lymphocyte, T-lymphocyte, NK cells, and monocyte markers. The unpaired t-test was carried out to evaluate the differences in these markers between T2DM patients and healthy individuals.

**Results:**

Patients with T2DM were associated with a lower percentage of total lymphocytes, higher percentage of B-lymphocytes, naive, and memory B subsets. In addition, patients with T2DM showed lower percentage of total T-lymphocytes (CD3+) and CD4 T-cells, but higher CD8 T-cell expression. Also, the NK-cell level was reduced in patients with T2DM, and the levels of monocyte subsets were altered.

**Conclusion:**

These data suggested that levels of lymphocytes and monocytes are impaired in T2DM patients, and this might be associated with the higher risk of infections observed in these patients.

**D**iabetes mellitus (DM) is a common metabolic disorder characterized by the presence of hyperglycemia and it is linked to various complications, including neuropathic, macro-vascular, and micro-vascular disorders.^1^ Diabetes mellitus is a global concern, given that the number of people with this disease has doubled in the last 3 decades.^2^ Worldwide, nearly 415 million people are currently diagnosed with DM, and the number is expected to be approximately 642 million by 2040.^3^ Diabetes mellitus is a key clinical and public health challenge in the Middle East region, with the prevalence of DM increasing in the Gulf Cooperation Council countries of Bahrain, Kuwait, Oman, Qatar, Saudi Arabia, and the United Arab Emirates.^3^ The situation is especially worrying in Saudi Arabia, where it has been claimed that 23.7% of Saudis have DM, with adult males experiencing a higher frequency than females.^1^

Diabetes mellitus is classified into 2 types; type 1 and 2, in which type 1 DM (T1DM) is a chronic autoimmune condition caused by pancreatic-cell death.^4^ In contrast, type 2 DM (T2DM) is a disease of increasingly impaired glucose regulation caused by a combination of malfunctioning pancreatic beta cells and insulin resistance.^5^

Type 2 DM is the most prevalent type of DM and has been progressively rising in frequency around the world, particularly in developing nations, accounting for 90-95% of all DM cases.^1,6^ It is linked to high rates of morbidity and mortality, which may have an impact on the overall health of patients as well as their quality of lifestyles.^1^ Type 2 DM affects people of all ages, including adolescents and young adults, however it is more common in adults.^1^

The key factors that contribute to T2DM development are genetics and lifestyle factors, including physical inactivity, a sedentary lifestyle, cigarette smoking, and excessive alcohol use.^7^ Research has shown that unhealthful eating habits and lifestyles cause insulin resistance in the body’s cells, which results in the development of T2DM.^1,6^ Additionally, over the past 20 years, obesity has been recognized as a significant risk factor for the emergence of T2DM and is linked to worse outcomes.^8^ In Saudi Arabia, economic growth has supported the adoption of luxurious lifestyles and, as a consequence, there is a tendency toward less physical activity and more unhealthy dietary habits.^6^ These factors have led to the increased prevalence of T2DM in Saudi Arabia, and actions should be taken to reduce the incidence of T2DM.^6^

Numerous studies have demonstrated that the immune system contributes to the development of T2DM.^9^ Additionally, the higher prevalence of infections in DM patients raises the possibility that DM has an impact on the immune system.^9^ Studies have shown that polymorphonuclear cells and monocytes from patients with DM have reduced functions (chemotaxis, phagocytosis, and killing) compared to cells from control groups.^9^ Based on CD14 and CD16 expression, monocytes can be classified into 3 subtypes: classical (CD14+/CD16-), intermediate (CD14+/CD16+), and non-classical (CD14-/CD16+).^10^ These subtypes differ in their traits and secrete various cytokines.^11^ It has been shown that people with T2DM have an altered monocyte phenotypes.^12^ Dysfunction of other immune cells, such as natural killer (NK) cells and neutrophils, has also been observed in cases of T2DM.^9^ In addition, total T-lymphocytes, CD4+, and CD8+ subsets were found to be associated with the development of T2DM, and that CD4+ and CD8+ T-cells are critical for the progression of DM in mice and humans.^13,14^ B-lymphocytes can be classified into many subtypes (based on CD27 and CD38 expression), including mature naive B-cells (CD27-/CD38+), plasmablasts (CD27+/CD38+), and resting memory B-cells (CD27+/CD38-).^15^ It should be noted that the few studies that have investigated the levels of B-lymphocyte subsets in patients with T2DM have shown that B-cells are activated in T2DM patients.^16,17^

Despite the fact that various studies have explored the phenotype of lymphocytes and monocytes, limited data are available regarding the phenotype of these cells in patients with T2DM in Taif, Saudi Arabia. Thus, the present study was designed to evaluate the levels of B-lymphocytes, T-lymphocytes, and NK cells and their subtypes in patients with T2DM and healthy individuals using flow cytometry. We also aimed to measure and compare the levels of B-lymphocyte subsets (based on CD27 and CD38 expression), T-lymphocyte subsets (CD4 helper and CD8 cytotoxic), and monocyte subsets (based on CD14 and CD16 markers) in patients with T2DM and healthy controls.

## Methods

In this case-control study, a total of 95 participants were recruited: 62 T2DM patients and 33 healthy controls. Patients with a confirmed diagnosis of T2DM were included in the study. Patients with incomplete data regarding age and gender were excluded from the study. The age range of the patients was 45-68 years old. Clinical data were obtained from hospital records. All the patients were admitted to the Diabetic Centre in Taif, Saudi Arabia. Blood samples were collected between April and August 2022. The hemoglobin A1c (HbA1c) level was measured and evaluated according to the guidelines of World Health Organization (WHO) using an automated analyzer (Bio-Rad, New York, NY, USA). Each participant in the study (patients and healthy controls) signed an informed consent form. The study was approved by the Research Ethics Committee at Taif University, Taif, Saudi Arabia (approval number: 43-133). The study was carried out according to the principles of the Helsinki declaration.

Blood samples from the T2DM patients and healthy individuals were collected in ethylenediaminetetraacetic acid (EDTA) tubes and processed within 24-48 hours after collection. By density centrifugation with a Ficoll-Paque gradient, peripheral blood mononuclear cells (PBMCs) were separated from the whole blood samples. For this, each whole blood sample was carefully layered onto one volume of Ficoll-Paque (GE Healthcare, Little, Chalfont, Buckinghamshire, UK) and centrifuged at 2,000 rpm for 20 minutes at 4^°^C with the brake off. The PBMCs were stained with different fluorophore-labelled antibodies, including CD3 (FITC), CD4 (APC), CD8 (PE), CD19 (FITC), CD27 (PerCp Cy5.5), CD38 (APC), CD56 (PE), CD14 (APC Cy7), and CD16 (FITC). First, cells were washed with phosphate buffer saline (PBS) and incubated with the appropriate antibodies at a 1:100 ratio for 30 minutes in the dark. The cells were then washed with PBS and resuspended in 200 μL of PBS. The samples were analyzed via a BD FACSCanto II system (BD Bioscience, San Jose, CA, USA) and FACSDiva software, version 6 (BD Biosciences). The lymphocytes and monocytes were gated according to the side scatter (SSC) and forward scatter (FSC). The data were analyzed via FlowJo software version 7.10 (Tree Star, Ashland, Oregon, USA).

### Statistical analysis

Data were analyzed as described previously.^18^ GraphPad Prism software, version 6.04 (La Jolla, CA, USA) was used to analyze the data. Patients’ data are shown as mean and standard deviation (SD). Differences in the lymphocyte and monocyte surface markers between the T2DM patients and healthy individuals were analyzed via an unpaired t-test or Mann-Whitney test according to the data distribution. Data were considered statistically significant if a *p*-value of <0.05 was detected.

## Results

The mean age of the patients enrolled in the study was 59±9.1 years. Gender diversity was achieved in this study with 53.2% of the patients being males and 46.8% being females. Table 1 provides a summary of the patients’ characteristics. Each patient’s HbA1c level was measured, and the resultant data shows that the mean glycemic index (HbA1c) was high (8.6±2.3%) among the patients. This indicates that the patients had uncontrolled levels of HbA1c.

**Table 1:** - Patient sample characteristics.

Parameters	T2DM	Healthy controls
Age (years), mean±SD	59±9.1	46±5.3
***Gender***		
MaleFemale	33 (53.2)29 (46.8)	19 (57.5)14 (42.5)
HbA1c (%), mean±SD	8.6±2.3	5.3±1.4

Values are presented as numbers and precentages (%) or mean ± standard deviation (SD). HbA1c: hemoglobin A1c, T2DM: type 2 diabetes mellitus

**Figure 1:**
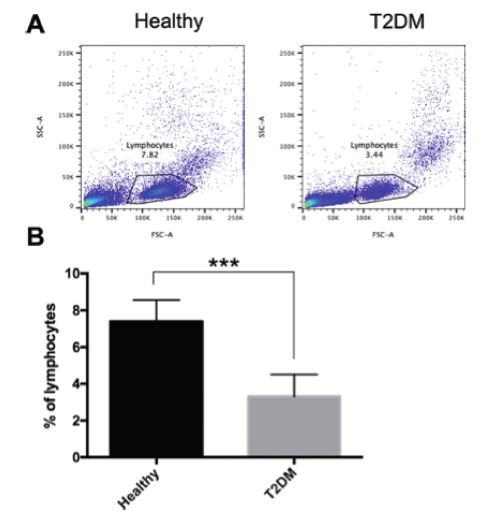
- Percentage of lymphocytes in T2DM patients and healthy individuals. Lymphocytes were isolated from the peripheral blood of the T2DM patients and healthy individuals based on SSC and FSC using flow cytometry. **A**) Dot plots showing the percentage of lymphocytes in the samples obtained from healthy individuals and T2DM patients. **B**) The percentage of lymphocytes in both groups is presented. Data were analyzed using an unpaired t-test. Values are presented as mean±SD. ^***^ *p*≤0.001, T2DM: type 2 diabetes mellitus, SSC: side scatter, FSC: forward scatter

The dot plots shown in Figure 1A demonstrate that the lymphocyte counts of the patients with T2DM were lower than those of the healthy individuals. In addition, the mean percentage of lymphocytes was found to be lower in the patients with T2DM compared with that in the control group, and the difference was statistically significant (*p*≤0.001, Figure 1B). This indicates that lymphocyte expression was impaired in the T2DM patients.

**Figure 2:**
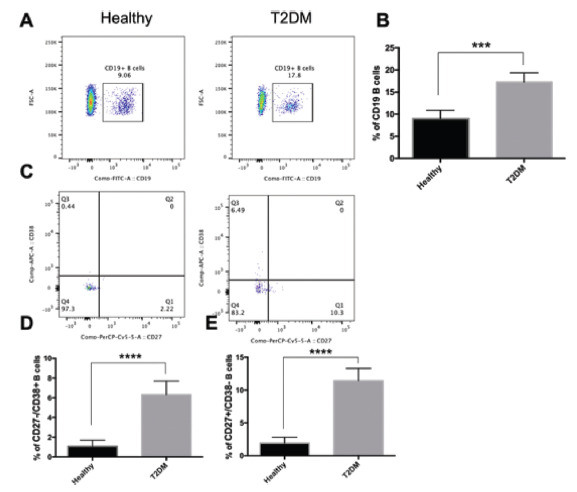
- Levels of B-lymphocytes and their subsets in T2DM patients and healthy individuals. Lymphocytes were isolated from the peripheral blood of the T2DM patients and healthy individuals. The cells were then stained with anti-CD19 (FITC), CD27 (PerCyp Cy5.5), and CD38 (APC) and analyzed using flow cytometry. **A**) Representative dot plots showing the percentage of CD19+ B-cells in the samples obtained from healthy individuals and T2DM patients. **B**) The percentage of CD19+ cells in both groups is presented. **C**) Representative dot plots showing the percentage of B-cell subsets in the samples obtained from healthy individuals and T2DM according to CD27 and CD38 expression. **D**) The percentage of CD27-/CD38+ cells in both groups is presented. **E**) The percentage of CD27+/CD38- cells in both groups is presented. Data were analyzed using an unpaired t-test. Values are presented as mean±SD. ^***^ *p*≤0.001, ^****^ *p*≤0.0001, T2DM: type 2 diabetes mellitus

To identify any differences in B-lymphocytes between the T2DM patients and healthy controls, we first examined the total B-lymphocytes (CD19+) using flow cytometry. As shown in Figure 2A, the patients with T2DM had higher expression of CD19+ B-lymphocytes than the healthy controls. The data indicate that the expression of CD19+ B-cells was twice as high in the patients with T2DM (17.5%) as in the healthy controls (9.1%) and that the difference was statistically significant (*p*≤0.001, Figure 2B). We were also interested in determining whether there were any differences in the levels of B-cell subsets. Subsets of B-lymphocytes were identified based on CD27 and CD38 expression and, as shown in the representative dot plots, there were different frequencies of B-cell subsets detected in the sample from the T2DM patients and the healthy controls (Figure 2C). The percentage of naive B-cells (CD27-/CD38+) was significantly higher in the patients with T2DM compared to that in the healthy controls (*p*≤0.0001, Figure 2D). Furthermore, the expression of resting memory B-cells (CD27+/CD38-) was significantly higher in the patients with T2DM compared to that in the control group (*p*≤0.0001, Figure 2E).

**Figure 3:**
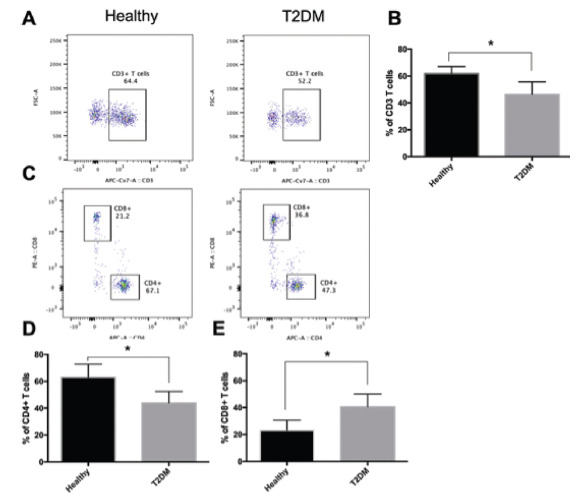
- Levels of T-lymphocytes and subsets in T2DM patients and healthy individuals. Lymphocytes were isolated from the peripheral blood of T2DM patients and healthy individuals. The cells were then stained with anti-CD3 (APC Cy7), CD4 (APC), and CD8 (PE) and analyzed using flow cytometry. **A**) Dot plots showing the percentage of CD3+ T-cells in the samples obtained from healthy individuals and T2DM patients. **B**) The percentage of CD3+ cells in both groups is presented. **C**) Dot plots showing the percentage of CD4 and CD8 T-cells in the samples obtained from healthy individuals and T2DM patients. **D**) The percentage of CD4+ cells; and **E**) CD8+ cells in both groups is presented. Data were analyzed using an unpaired t-test. Values are presented as mean±SD. ^*^ *p*≤0.05, T2DM: type 2 diabetes mellitus

Initially, the total T-lymphocyte (CD3+) levels were examined using flow cytometry. As shown in Figure 3A, the patients with T2DM had lower expression of CD3+ T-lymphocytes compared to the healthy controls. It was also found that the patients with T2DM had a significantly lower mean percentage of CD3+ T-lymphocytes compared to the healthy controls (*p*=0.012, Figure 3B). The percentage of T-lymphocytes was 40% in the T2DM patients and 60% in the healthy controls. After determining that the total T-lymphocyte count was different in the T2DM group compared to that in the healthy group, we examined the expression of T-cell subsets (CD4 and CD8) using flow cytometry. The numbers and proportions of CD4 and CD8 T-cells detected in the samples from the T2DM patients and healthy controls are indicated by the representative dot plots shown in Figure 3C. It was found that CD4 expression was significantly lower in the T2DM patients compared to that in the healthy controls (*p*=0.011, Figure 3D). In contrast, CD8 expression was significantly higher in the T2DM patients compared to that in the control group (*p*=0.012, Figure 3E).

**Figure 4:**
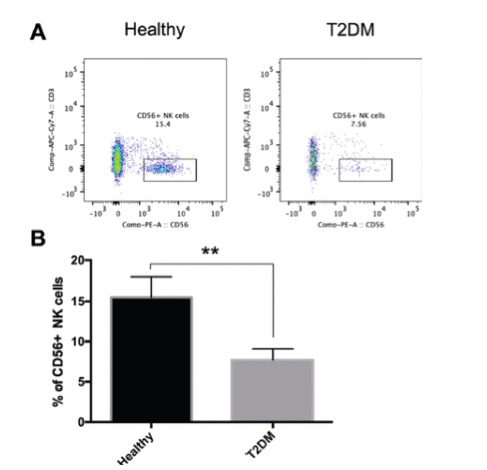
- Levels of NK cells in T2DM patients and healthy individuals. Lymphocytes were isolated from the peripheral blood of the T2DM patients and healthy individuals. The cells were then stained with anti-CD3 (APC Cy7), and CD56 (PE) and analyzed using flow cytometry. **A**) Dot plots showing the percentage of CD3-/CD56+ NK cells in the samples obtained from healthy individuals and T2DM patients. **B**) The percentage of NK cells in both groups is presented. Data were analyzed using an unpaired t-test. Values are presented as mean±SD. ^**^ *p*≤0.01, T2DM: type 2 diabetes mellitus

We also investigated the levels of NK cells (CD3-/CD56+). As shown in Figure 4A, the T2DM patients had lower levels of NK cells than the healthy controls. This difference was found to be statistically significant (*p*=0.009, Figure 4B).

**Figure 5:**
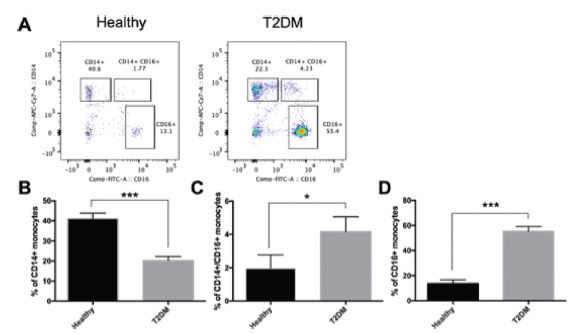
- Expression of monocyte subsets in T2DM patients and healthy individuals. Monocytes were isolated from the peripheral blood of the T2DM patients and healthy individuals. The cells were then stained with anti-CD14 (APC Cy7), and CD16 (FITC) and analyzed using flow cytometry. **A**) Dot plots showing the percentage of monocyte subsets in the samples obtained from healthy individuals and T2DM patients. **B**) The percentage of CD14+ cells; **C**) CD14+/CD16+; and **D**) CD16+ in both groups are presented. Data were analyzed using an unpaired t-test. Values are presented as mean±SD. ^*^ *p*≤0.05, ^***^ *p*≤0.001. T2DM: type 2 diabetes mellitus

We evaluated the levels of monocyte subsets according to the expression of CD14 and CD16. As shown in Figure 5A, the monocyte phenotype varied among the T2DM patients and the healthy controls. The expression of classical monocytes (CD14+/CD16-) was significantly reduced in the T2DM patients compared with that in the control group (*p*=0.0005, Figure 5B). However, the expression of intermediate monocytes (CD14+/CD16+) was significantly higher in the T2DM patients than in the control group (*p*=0.011, Figure 5C). The patients with T2DM were found to have significantly higher expression of non-classical monocytes (CD14-/CD16+) compared to the control group (*p*=0.0001, Figure 5D).

## Discussion

The present study was designed to evaluate the levels of total lymphocytes, B-lymphocytes (CD19+), T-lymphocytes (CD3+), NK cells, and monocyte subsets in patients with T2DM in Saudi Arabia and compare them to those found in healthy individuals. We also aimed to evaluate whether the levels of B- and T-lymphocyte subsets are frequently altered in patients with T2DM. Alterations in the levels of these important immune cells can be used to predict the severity of disease progression in T2DM patients.^9^ First, we have shown that the percentage of total lymphocytes was severely reduced in patients with T2DM compared to healthy individuals. Type 2 DM patients with lower lymphocyte counts have an increased risk of infection, and the ability of immune cells to respond to challenge has been found to be decreased in subjects with T2DM.^9,19^ However, another study found no difference in the percentage of lymphocytes in patients with T2DM.^13^ In this particular study, the patients had normal HbA1c levels, suggesting that patients with controlled levels of HbA1c would have no impact on lymphocyte levels.

Although the percentage of total lymphocytes was lower in our cohort of T2DM patients compared to the healthy controls, the levels of B-lymphocytes were higher in the T2DM patients compared to the healthy controls. In alignment with our results, a previous study showed that the B-lymphocyte level was higher in patients with T2DM, especially those with obesity.^16,17^ Regarding the phenotype of B-lymphocyte subsets in patients with T2DM, there are limited reports in the literature on this topic. In the present study, the patients with T2DM displayed different proportions of B-lymphocyte subsets to healthy individuals, with the percentage of naive (CD27-/CD38+) and memory (CD27+/CD38-) B-cells found to be elevated in patients with T2DM. Our findings are consistent with those of previous studies that indicated that the levels of naive and memory B-cells were higher in T2DM patients compared to those in healthy people.^17^ It should be noted that different strategies have been implemented to define B-lymphocyte subsets and have failed to provide consistent results.^20^ A study in which B-cell subsets were defined solely by CD27 expression found that naive and memory B-cell expression was lower in patients with T2DM.^16^ Another study defined B-cell subsets according to CD38 and CD24 expression, and our findings are consistent with the results of that study.^17^ It has also been suggested that 10 B-cell subsets can be defined by using a panel of different flow cytometric markers, including CD27, CD24, CD38, CD5, CD10, CD21, and CD22.^21^

Our findings indicate that the percentage of total T-lymphocytes (CD3+) was lower in the patients with T2DM compared with the healthy controls. This aligns with another study that reported that T-lymphocytes declined in patients with T2DM.^22^ To examine this finding in more depth, we evaluated the expression of helper (CD4+) and cytotoxic (CD8+) T-cells in patients with T2DM. Our data suggest that CD4+ expression was lower and that CD8 expression was higher in the T2DM patients than in the healthy individuals. Inconsistent results have been reported regarding the expression of CD4 and CD8 in patients with T2DM. In one study, no differences in the levels of CD4 and CD8 in patients with T2DM and healthy individuals were observed.^13^ The findings of another study suggested that the levels of both CD4 and CD8 were higher in T2DM patients than in healthy individuals.^23^ Furthermore, CD4 and CD8 T-cell phenotyping analysis carried out in yet another study showed that there was a significant decrease in both CD4 and CD8 in T2DM patients compared to healthy individuals.^24^ Nevertheless, our findings suggest that the T-lymphocyte response is impaired in patients with T2DM.

In this study, NK cell counts in T2DM patients were found to be lower than in healthy controls. Various studies have evaluated the levels of NK cells in patients with T2DM.^25,26^ Our findings are consistent with those of a study that reported a reduction in NK-cell expression.^25^ In that study, the level of NK cells was found to be associated with the level of HbA1c. Natural killer cells release important cytokines, such as interferon-gamma and tissue necrosis factor-alpha; therefore, a lower level of NK cells might contribute to an increased susceptibility to infection.^25^ However, another study found that NK-cell expression was higher in T2DM patients than in healthy controls.^26^

Finally, we evaluated the levels of monocytes in the patients with T2DM. The patients with T2DM had lower levels of classical monocytes and higher levels of both intermediate and non-classical monocytes. A previous study also found that the levels of classical monocytes were reduced.^11^ Consistent with our findings, a recent systematic review found that the expression of intermediate and non-classical monocytes was higher in T2DM patients.^27^ It has been suggested that the levels of non-classical monocytes are elevated during infection and inflammation and are associated with a poor glycemic index in patients with T2DM.^28,29^

### Study limitations

First, the sample size was relatively small. Second, the B-lymphocyte subsets were defined according to only 2 surface markers (CD27 and CD38). It is recommended that future studies include a larger B-lymphocyte panel.

In conclusion, we assessed the levels of lymphocytes and monocytes in patients with T2DM in Taif, Saudi Arabia, and found that their phenotypes were altered. Patients with T2DM were found to have lower percentages of total lymphocytes and higher expression of B-lymphocytes and naive and memory B-cell subsets compared to healthy individuals. In addition, CD4+ T-cell expression was lower and CD8+ T-cell expression was higher in the T2DM patients. The levels of NK cells were reduced in the patients with T2DM. Furthermore, the patients with T2DM also had lower levels of classical monocytes and higher levels of both intermediate and non-classical monocytes. These data suggest that the levels of B-lymphocytes, T-lymphocytes, NK cells, and monocyte subsets are impaired in patients with T2DM in Taif, Saudi Arabia, and this might be associated with the higher risk of infections observed in these patients.
